# 

**Published:** 1891-12

**Authors:** 


### CONTENTS FOR DECEMBER, 1891.

ORIGINAL COMMUNICATIONS.

Report of Cases of Appendicitis, with Remarks By JOSEPH PRICE, M. D. .... 157
Four Operations for Appendicitis: Three Recoveries; One Death from a very Small Concealed Abscess. By W. W. KEEN, M. D. .... 269
The Operative Treatment of Appendicitis. By THOMAS S. K. MORTON, M. D. .... 275

EDITORIAL.

The Southern Surgical and Gynecological Association .... 300
The Empire State Express .... 302
The Appendicitis Discussion of the Philadelphia County Medical Society .... 303

PERSONAL.

Dr. Ernest Wende—Dr. W. E. B. Davis—Prof. W. T. Briggs .... 304
Dr. C. A. L. Reed—Dr. John A. Pettit—Dr. Wm. A. Wheeler .... 305

JOURNALISTIC NOTES.

New York *Journal of Gynecology and Obstetrics—American Gynecological Journal* .... 305

SOCIETY MEETINGS.

The Buffalo Clinical Society—The Eighty-sixth Annual Meeting of the Medical Society of the State of New York .... 306

BOOK REVIEWS.

On the Medical and Surgical Uses of Electricity .... 307
Insomnia and its Therapeutics .... 309
Surgery: A Practical Treatise with Special Reference to Treatment .... 310
Practical Treatise on Electricity in Gynecology .... 311
The *Medical News* Visiting List for 1892 .... 312
Stories of a Country Doctor .... 313
Syllabus of the Obstetrical Lectures in the Medical Department of the University of Pennsylvania .... 313
Essentials of Anatomy and Manual of Practical Dissection .... 314
The Genuine Works of Hippocrates .... 315
Chemical Lecture Notes .... 315
The Physician's Visiting List (Lindsay & Blakiston's) for 1892 .... 315
Physician's Leisure Library .... 316
Massage: A Primer for Nurses .... 316
Books and Pamphlets Received .... 317

MISCELLANY.

Paraffine in Diphtheria .... 299
The *Obstetrical Record* .... 319
All Around the Year 1892 .... 320

